# Supplementary material for: Comparative multiomics analysis of cell physiological state after culture in a basket bioreactor
Source: Sci Rep. 2022 Nov 23;12:20161. doi: 10.1038/s41598-022-24687-4 (PMC9686226; doi:10.1038/s41598-022-24687-4)
Supplement: Supplementary file 1 — Supplementary Information 1. [file 41598_2022_24687_MOESM1_ESM.zip › raw data/Proteomic raw data/6.KEGGenrichment/EG.vs.CG/DOWN/pathway/src/05152.html]

map05152


- K06560

- Down regulated proteins

A0A0D9QV27(Description:Uncharacterized protein;Gene:MRC2;log2FC:-1.0376)

- K01379

- Down regulated proteins

A0A0D9RBZ8(Description:Cathepsin D;Gene:;log2FC:-1.4696)

- K06462

- Down regulated proteins

A0A0D9QZZ6(Description:VWFA domain-containing protein;Gene:ITGAX;log2FC:-1.9952)

Close
